# Effects of power ultrasound treatment on the shelf life of button mushrooms: Digital image processing and microbial counting can reveal the effects

**DOI:** 10.1002/fsn3.2303

**Published:** 2021-05-07

**Authors:** Maryam Ganjdoost, Mohammad Aboonajmi, Hossein Mirsaeedghazi, Keyvan Asefpour Vakilian

**Affiliations:** ^1^ Department of Agrotechnology College of Abouraihan University of Tehran Tehran Iran; ^2^ Department of Food Technology College of Abouraihan University of Tehran Tehran Iran; ^3^ Department of Biosystems Engineering Gorgan University of Agricultural Sciences and Natural Resources Gorgan Iran

**Keywords:** artificial neural networks, button mushroom, Image processing, storage, ultrasound

## Abstract

Edible button mushroom (*Agaricus bisporus*) is the most common commercial‐grade mushroom in the world. The shelf life of button mushrooms is limited to a range between two and four days because of enzymatic browning at medium ambient temperature if it is minimally processed. This study aimed to investigate the effects of power ultrasonics and its interaction with several treatments including H_2_O_2_ and O_3_ on increasing the storage quality of edible button mushroom by controlling enzymatic browning. A 100 W ultrasonic bath with a frequency between 20 and 35 kHz was used during the experiments. The storage quality was studied by examining the changes in color and microbial content over 12 days. The results obtained from the digital image processing and total microbial counting showed that the ultrasonic treatment for 6 min is an appropriate method for controlling the color preservation of mushrooms to improve their shelf life. The maximum changes in RGB band, HSV band, L*a*b* band, and microbial content of the mushroom samples under the ultrasonic treatment were equal to 7%, 12%, 10%, and 11%, respectively. Furthermore, having the color properties and microbial content of the samples, the artificial neural network (ANN) was capable of predicting their storage period with an *MSE* of 0.011.

## Graphical abstract

This study aimed to investigate the effects of power ultrasonics and its interaction with several treatments including H2O2 and O3 on increasing the storage quality of edible button mushroom by controlling enzymatic browning. The results obtained from the digital image processing and total microbial counting showed that the ultrasonic treatment for 6 min is an appropriate method for controlling the color preservation of mushrooms to improve their shelf life.

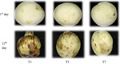

## 1 INTRODUCTION

Button mushroom (*Agaricus bisporus*) is widely cultivated throughout the world and has a special place in the food basket of the people due to its good nutritional quality (Ramos et al., 2019). *A. bisporus* is a valuable food with high amounts of amino acids, vitamins, minerals, polyphenol oxidases, proteins, and dietary fiber (Kalač, 2013; Muszyńska et al., 2017; Rathore et al., 2017). It has a higher respiration rate than other fruits and vegetables, and due to the lack of a protective layer to prevent water loss, it rots faster, limiting its shelf life in the chain of distribution and supply (Ares et al., 2006; Singh et al., 2010).

The most important indicators of the quality of button mushrooms are its whiteness, round and glossy cap, straight stem, and lack of brown spots (Singh et al., 2010). The mushroom begins to rot the day after harvest, starting with discoloration and turns from white to brown due to the activity of the tyrosinase (Nerya et al., 2006). The shelf life of button mushrooms is from 2 to 4 days at an average storage temperature (Ares et al., 2006), which this short shelf life is the most important constraint in the industrial production of *A. bisporus*. The shelf life of the mushrooms is limited by the enzymatic browning if they undergo minimal processing. These browning reactions are related to mechanical damage during transport, processing, scratching, washing, aging, and bacterial infections, and reduce the quality of the processed products. In unsuitable storage conditions, the button mushroom begins to lose weight (about 5%–10%) and becomes unusable. Therefore, its postharvest physiology needs further study due to problems in the distribution and market of fresh mushrooms (Ares et al., 2006). Hence, it is necessary to use appropriate methods to increase postharvest shelf life and maintain the quality of *A. bisporus*.

In recent years, various methods have been developed for the postharvest quality improvement of *A. bisporus*, such as physical treatments (e.g., coating), radiation, modified atmosphere, special packaging, and the use of nanotubes (Zalewska et al., 2018). Products such as mushrooms are more sensitive to other crops due to the lack of a protective layer against microbial attack and pests. After harvesting, the rate of decay of mushrooms depends on their initial microbial content. The Gram‐negative *Pseudomonas tolassii* microorganism, as a natural constituent of mushroom growing soil, can produce a toxic metabolite in the fungus under certain conditions, which appears as a brown injury to the product. Other Gram‐negative microorganisms such as *Pseudomonas fluorescens* and yeasts affect the product rotting. Molds can also affect the quality of mushrooms: Infection with *Verticillium maltose* also causes brown spots (Fernandes et al., 2012). Therefore, it is necessary to use modern methods for noncontact washing as well as complete microbial disinfection.

The use of ultrasound improves the quality of the washed product due to the complete removal of manual washing. This method, in combination with various disinfectants including O_3_ washing, can be used as a novel method to improve the shelf life of agricultural and horticultural products (Chen & Zhu, 2011). Ultrasound can be used as a nondestructive method to increase the storage quality of garden and greenhouse products such as mushrooms and strawberries (Li et al., 2017). Washing is carried out fast and with high accuracy in this method. Washing time in this method is almost 3, 6, up to 20 min depending on the type of washing, while conventional washing is time‐consuming. Ultrasonic pretreatment involves immersing the fruit in distilled water or hypertonic aqueous solution when applying ultrasonication. Ultrasound causes a series of rapid contractions and intermittent expansions (Fernandes and Rodrigues, 2008). Furthermore, ultrasonication causes cavitation within the food material which may be useful for separating the attached water (Soria and Villamiel, 2010). Aday et al. (2013) reported the use of ultrasound as an innovative method to extend the strawberry's shelf life, pH changes, soluble solid content, color, mold growth, and texture. Ultrasound is one of the newest nondestructive methods that extend the life span of fresh fruits during storage. They studied different ultrasonic powers (30, 60, and 90 W) and different treatment times (5 and 10 min) on strawberry quality and showed that the effectiveness of ultrasound depends on the frequency, wave power, time, and temperature during the experiment.

In another study, Lagnika et al. (2013) examined the extension of the life of mushroom by applying high‐pressure argon during storage. The effects of ultrasound, high‐pressure argon, and their interactions on the physicochemical and microbiological characteristics of white button mushrooms were studied for 9 days at the storage temperature of 4°C. High‐pressure argon treatment was relatively effective in maintaining the firmness and was suitable for maintaining cell integrity. Gao et al. (2014) investigated the effects of essential oils on preventing browning of product quality parameters and reducing the quality of button mushroom. They showed the highest percentage of browning in the control samples and better quality in samples affected by the essential oils of clove, cinnamon, and thyme during a 16 day storage period.

In recent years, machine learning has been widely used to model multi‐input problems in food technology (Erban et al., 2019; Schroeder et al., 2019). Neuron‐based and kernel‐based machine learning algorithms can reveal the nonlinear relationships between the inputs and the output (Amanabadi et al., 2019; Asefpour Vakilian, 2020). Artificial neural networks (ANNs) are one of these algorithms that can be efficiently used for food science and technology. Instead of cost‐ and time‐consuming laboratory experiments, the trained machines can reliably predict the attributes of the food samples. Estelles‐Lopez et al. (2017) used the machine learning method to predict the counts of microorganisms responsible for meat spoilage regardless of the packaging system applied. Barbon et al. (2016) predicted the storage time of pork using machine learning by having the pH, water holding capacity, color, and lipid oxidation extracted from samples of 0, 7, and 14 days of postmortem. Four variables, that is, pH, L*, a*, and b* were found to be useful in pork storage time determination.

At present, the effect of postharvest quality improvement methods on the appearance of the white button mushroom is determined visually using naked eyes by measuring the percentage of the brown surface of the fungus. This method seems to be inaccurate and cannot be used to determine the intensity of the brown surface of the product. To address this limitation, digital image processing can be a promising approach to accurately quantify the effects of postharvest improvement methods and treatments on its appearance and marketability. Currently, in some researches, digital image processing has been used to assess the storage quality of several agricultural products (Hu et al., 2016; Maniwara et al., 2014; Saeys et al., 2019). Therefore, this study aims to measure both color properties and microbial content of button mushrooms during 12 day storage at 4°C under power ultrasonics treatment and its interaction with other treatments to provide an efficient treatment for increasing the storage quality of this product. Furthermore, the performance of ANN as a robust and reliable machine learning algorithm was investigated to predict the storage time of mushroom samples based on their color properties and microbial contents.

## 2 MATERIALS AND METHODS

### 2.1 Mushroom material

**FIGURE 1:**
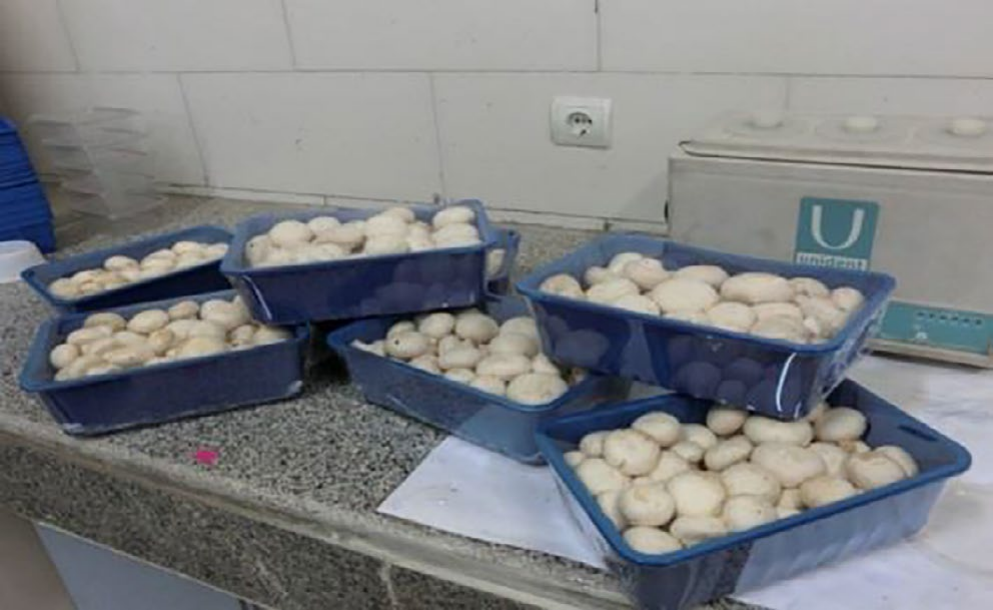
The study white button mushrooms

In this study, white button mushroom (*Agaricus bisporus*) was purchased from the Varna mushroom cultivation center in Tehran and transferred to a research laboratory in College of Abouraihan, University on Tehran. The mushrooms were relatively similar in size and free of physical damage and fungal infections (Figure 1). The samples were then immersed in distilled water to remove dust and external factors and then kept at room temperature and humidity for 1 hr to be dried and lose excess moisture. They were then placed in packages and kept at 4°C for 12 hr to relieve the initial stress.

### 2.2 Ultrasound system specifications

**FIGURE 2:**
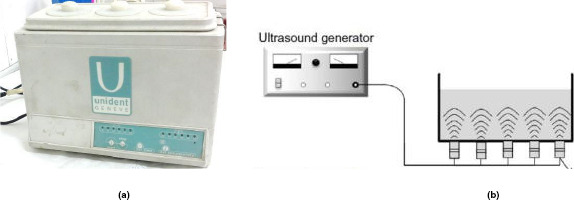
(a) Ultrasonic bath apparatus used in this research (b) Schematic view of the apparatus

An ultrasonic bath with an output power of 100 W and variable frequency, between 20 and 35 kHz, was used to provide power ultrasound (Figure 2). The ultrasound was applied in two levels of time duration: 4 and 6 min at the constant temperature of at 20°C.

### 2.3 O_3_ generator

**FIGURE 3:**
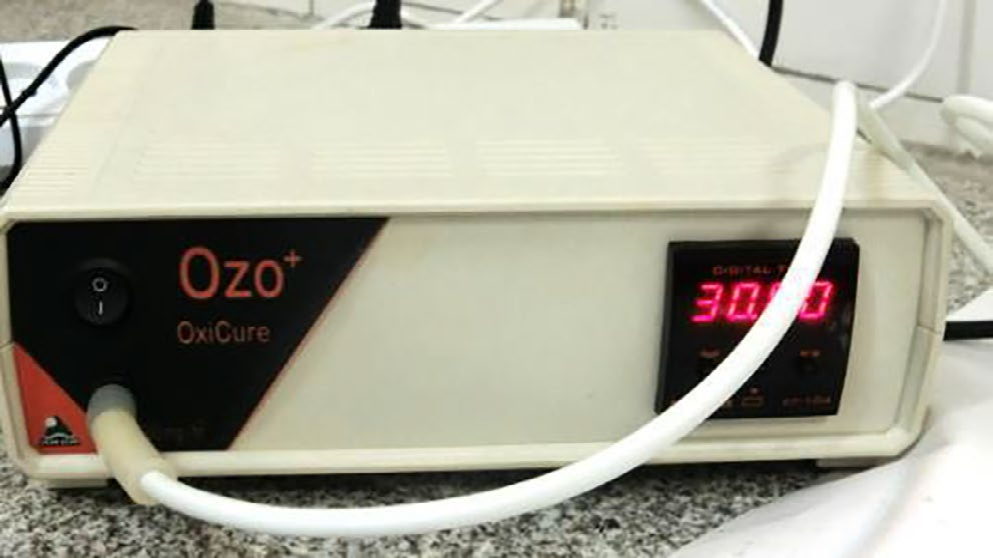
O_3_ generator model ARDA

An O_3_ generator was used to produce O_3_ (Figure 3). The amount of O_3_ produced by this device was 200 mg/h without an oxygen capsule.

### 2.4 Image processing chamber

**FIGURE 4:**
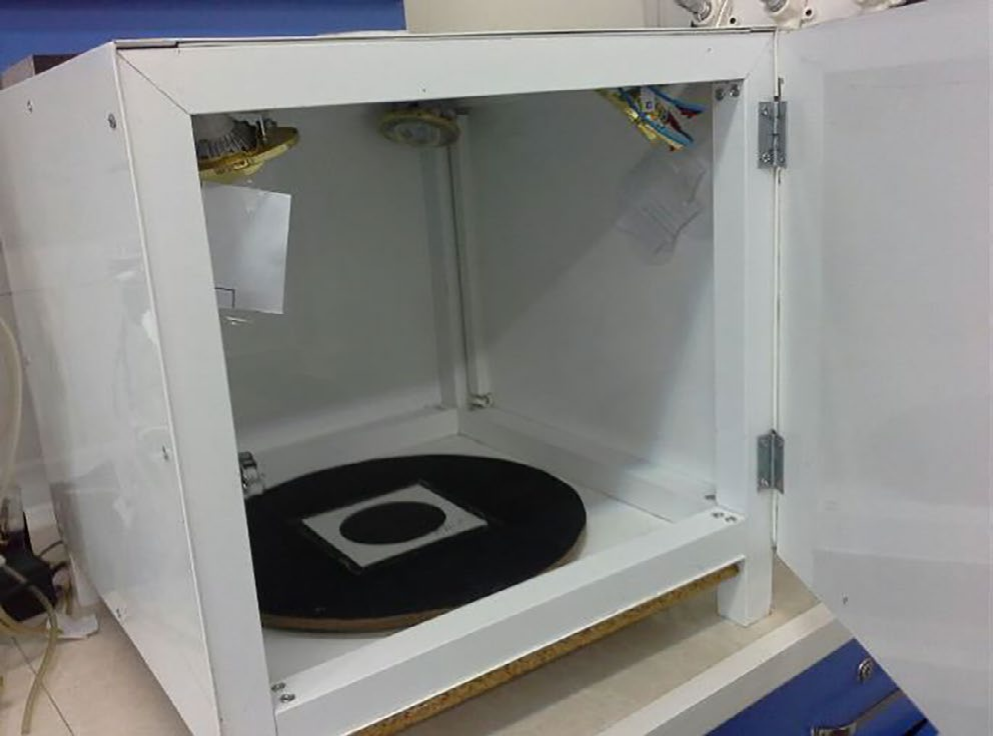
The image processing chamber used for collecting the data required for digital image processing

The image processing unit was a chamber with 55, 25, and 44 cm in length, width, and height, respectively, for the data collection required for the digital processing of the images (Figure 4). The chamber was equipped with four halogen lamps providing uniform illumination for imaging. There was a hole in the center of the upper side of the chamber for the camera lens to be placed completely vertically.

### 2.5 Color models

Three color models, that is, RGB, HSV, and L*a*b* were used to compare the effects of the treatments on the appearance of the samples. An RGB color image includes an array of *m* × *n* pixels, each of which has three red, green, and blue color bands. The HSV color model is defined by human perception and understanding of the concept of color. This color model is very similar to the color cylinder. In this cylinder, the main colors are placed surrounding the circumference of the circle, and the color intensity decreases from the circumference to the center of the circle, and the color brightness decreases from top to bottom. In the L*a*b* model, colors are characterized by three parameters: Luminance (L*); color change from green to red (a*); and color change from blue to yellow (b*). L* ranges from 0 (black) to 100 (white), a* from −120 (red) to +120 (green), and b* from −120 (blue) to +120 (yellow).

**FIGURE 5:**
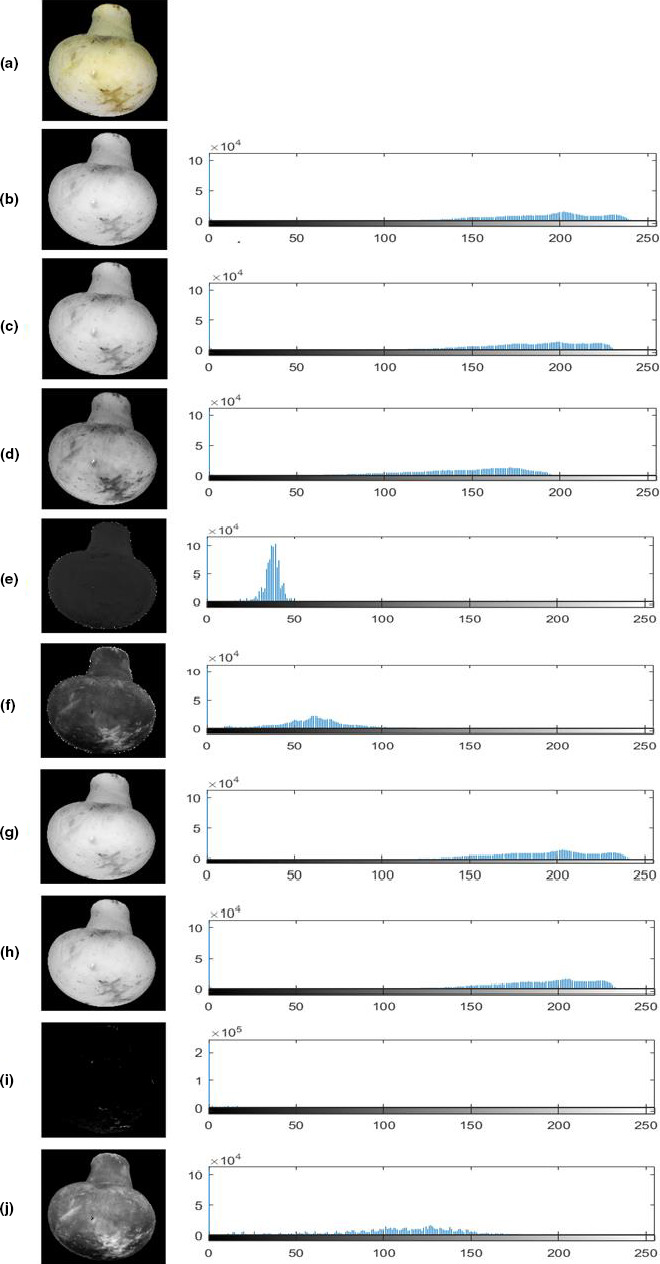
(a) Recorded image of an arbitrary button mushroom and normalized histogram graphs of image color bands, (b) R band, (c) G band, (d) B band, (e) H band, (f) S band, (g) V band, (h) L band, (i) a band, (j) b band

Figure 5 shows the color bands of an image of an arbitrary button mushroom which was captured using the image processing chamber. It can be seen that in each band, there might be useful information about the sample that does not exist in other bands. A histogram of each band is also shown in this figure. The horizontal axis of all bands is normalized between zero and 255 to determine the density of pixels gray‐level values. As can be seen, the a* band of the L*a*b* color model had almost no gray‐level values.

### 2.6 Total microbial counting

To determine the microbial content on the surface of the mushroom samples, total microbial counting was performed in the first, sixth, and twelfth days of the storage period. The culture medium used in the experiment was nutrient agar. The microbial content, in colony‐forming unit per gram (CFU/g), was measured by removing 1 g of the mushroom surface with a sharp razor, followed by homogenizing in a porcelain mortar. The sample was poured into a test tube containing 9 ml of distilled water and thoroughly homogenized with a mixer. A drop of the prepared suspension was evenly distributed in the nutrient agar medium and shaken slowly to harden. The tube was then kept in a growth chamber at 25°C for 24 hr, and then, the microbial content was counted by a colony counter (Sarlak et al., 2016).

### 2.7 Statistical analysis

The treatments performed in this study and their codes, from T1 to T9, are defined in Table 1. Experiments were based on completely randomized factorial design, and the *t* test was used to determine the difference between the treatments and the control using MATLAB R2018b programming environment.

**TABLE 1:** The codes for the treatments used in this study

Treatment	Code
Control	T1
Treating with distilled water for 4 min	T2
Treating with distilled water for 6 min	T3
Treating with H_2_O_2_ for 6 min	T4
Treating with O_3_ for 6 min	T5
Treating with ultrasonics in distilled water for 4 min	T6
Treating with ultrasonics in distilled water for 6 min	T7
Treating with ultrasonics in H_2_O_2_ for 6 min	T8
Treating with ultrasonics in O_3_ for 6 min	T9

### 2.8 Artificial neural network architecture

In this study, ANN as a machine learning method was used to predict the storage period of the mushrooms using their color values and total microbial content. A multi‐layer perceptron (MLP) was implemented, which is a class of feed‐forward ANN and is appropriate for prediction or approximation problems. The MLP network architecture with one hidden layer, which was used in this study, had nine inputs, namely three RGB bands, three HSV bands, two L* and b* bands, and one total microbial content features. One of the most significant parameters of an ANN is the number of neurons in the hidden layer. To find the optimum network, the number of neurons in the hidden layer was increased from 5 to 20 with increments of 2, and then, the performance of the network was assessed using the network error. The back‐propagation method was used for supervised learning of ANN. To minimize the errors, the Levenberg–Marquardt algorithm was used in the back‐propagation method. The performance of the model was assessed based on mean square error (*MSE*) (Equation 1)

(1) $$MSE=\frac{\sum_{i+1}^{n}{\left(X_{p}‐X_{o}\right)}^{2}\sum_{i=1}^{n}{\left(x_{p}‐x_{o}\right)}^{2}}{n}$$

where *X*_o_ and *X*_p_ are the observed and predicted values using machine learning, respectively, and *n* is the number of the data. The lower the *MSE*, the better performance of the machine learning model is.

## 3 RESULTS

### 3.1 Results of the image processing

The changes in the mean of gray‐level values of the image pixels from the studied button mushrooms during 12 days of storage were calculated using image processing in three color spaces of RGB, HSV, and L*a*b*. Table 2 shows the mean and standard deviation values of the color intensity at each band during the storage period. Since the values of a* band in the L*a*b* color model were negligible in all images captured by the image processing unit, this band is not shown in the table. In this table, the values of the treatments that showed a significant difference (*p* < .05) with the control for a given band in the *t* test are shown in bold. This table shows that treatments including ultrasonics significantly changed some of the color properties of the samples compared to those of the control.

**TABLE 2:** Mean (*μ*) and standard deviation (*σ*) of color intensities and total microbial content in different treatments

							Color properties										Total microbial content	
Treatment Code	RGB model						HSV model						L*a*b* model					
	R		G		B		H		S		V		L		b			
	*μ*	*σ*	*μ*	*σ*	*μ*	*σ*	*μ*	*σ*	*μ*	*σ*	*μ*	*σ*	*μ*	*σ*	*μ*	*σ*	*μ*	*σ*
T1	174.73	17.65	163.86	23.29	117.74	29.92	0.143	0.011	0.352	0.112	0.729	0.055	66.70	8.42	22.16	6.01	133.50	93.88
T2	175.80	13.26	166.18	17.20	118.31	24.58	0.141	0.007	0.344	0.091	0.690	0.053	67.55	6.17	24.30	6.51	176.17	41.80
T3	182.98	8.91	175.40	10.47	133.17	14.96	0.143	0.008	0.295	0.046	0.718	0.035	70.84	3.73	20.98	6.54	150.00	24.76
T4	**188.22**	**5.38**	**183.80**	**8.24**	**137.43**	**12.85**	0.147	0.005	0.293	0.060	**0.749**	**0.028**	**73.85**	**2.96**	22.58	7.71	**66.67**	**27.25**
T5	**183.23**	**9.39**	177.31	10.76	**137.88**	**11.67**	0.146	0.005	0.306	0.083	0.719	0.037	71.49	3.84	26.09	6.43	**72.00**	**20.42**
T6	**185.26**	**5.92**	**177.70**	**9.73**	132.76	17.08	0.146	0.010	0.299	0.080	0.727	0.021	71.68	3.41	23.38	6.67	**106.50**	**34.06**
T7	***182.58***	***5.08***	***184.11***	***5.71***	***141.94***	***11.30***	*0.143*	*0.004*	***0.263***	***0.043***	***0.744***	***0.020***	***73.97***	***2.07***	*25.47*	*6.92*	***99.00***	***4.44***
T8	181.58	13.95	177.93	17.28	135.92	22.62	0.138	0.011	0.289	0.091	0.685	0.070	**72.03**	**6.67**	23.40	6.33	**76.50**	**13.81**
T9	181.07	12.46	170.97	13.82	127.86	18.75	**0.149**	**0.006**	**0.263**	**0.057**	0.704	0.041	69.26	4.96	24.01	5.88	**83.83**	**44.17**

Note

Values that showed a significant difference (*p* < .05) with the control in the *t* test are shown in bold. The values with the lowest standard deviations for the studied attributes are shown in italic.

The treatments with the lowest standard deviations for the studied attributes are shown in italic. Comparing the standard deviation values of the studied attributes shows that, in general, the standard deviation values of the color intensity during the ultrasonic treatment with distilled water for 6 min was smaller than that of other treatments. This indicates that during the 12 day storage of the mushroom samples treated with sonication and distilled water, the changes of the color intensity of the samples were lower than that in other treatments. Browning of the samples during the storage increases the standard deviation of the product's color bands’ intensity. A zero value for the standard deviation reveals that the samples have not undergone any changes during the 12 days of storage which is the most desirable. Table 2 shows that compared to H_2_O_2_ and O_3_ treatments, sonication had acceptable performance in reducing the standard deviation of gray‐level values of image color bands.

**FIGURE 6:**
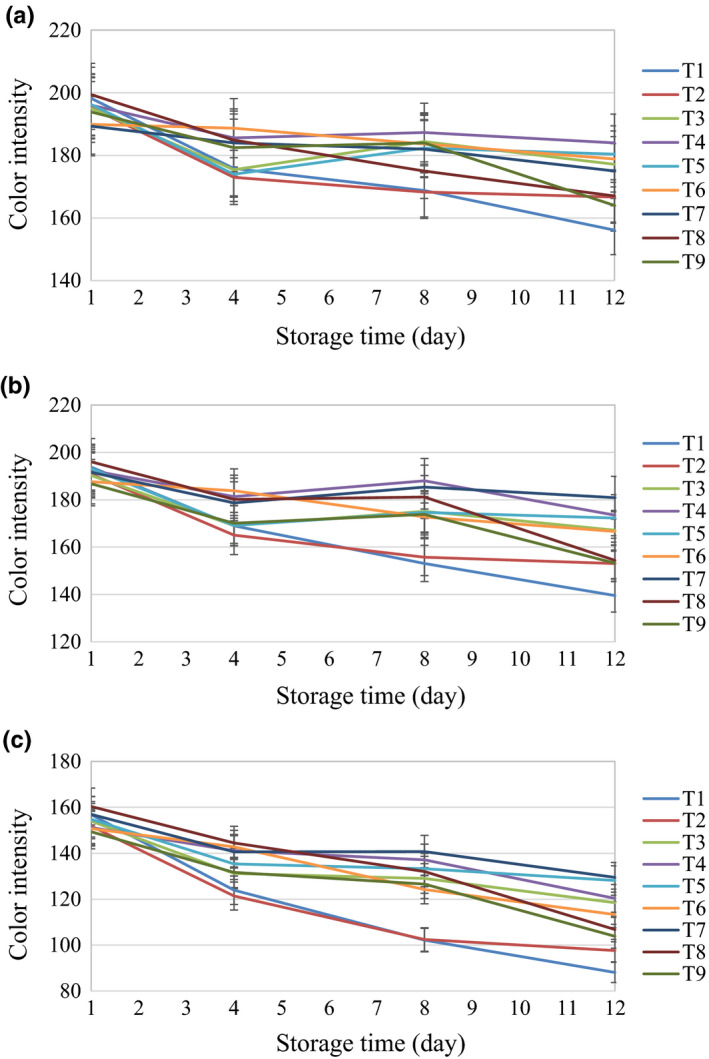
The changes in RGB color space properties during the storage time (a) R band, (b) G band, (c) B band

As shown in Figure 6, the effects of treatments on mushrooms during the 12 day storage period on the mean in R band in the RGB color space indicates that samples washed with distilled water, and H_2_O_2_ and O_3_ treatments for 6 min had similar results compared with control. However, treatments including ultrasonics were different from other treatments. This trend was almost similar for G and B bands, which confirms the results of the *t* test.

**FIGURE 7:**
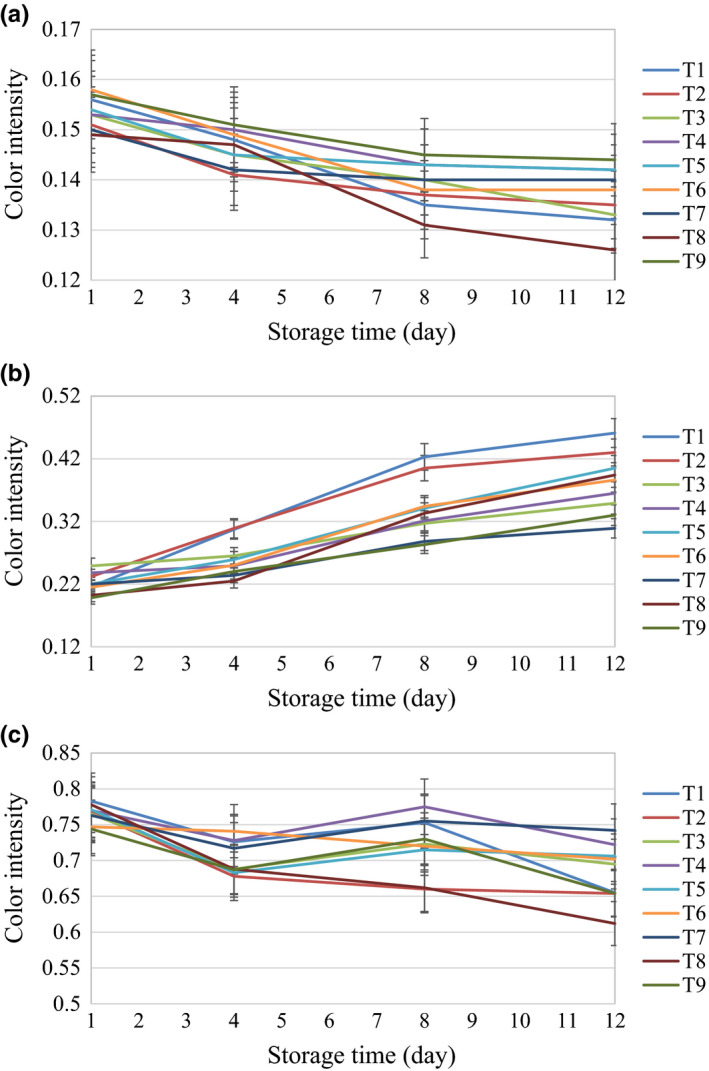
The changes in HSV color space properties during the storage time (a) H band, (b) S band, (c) V band

As shown in Figure 7, the trend of mean values of the H band over the 12 days of the storage indicates that from the first to the eighth day, all treatments had similar trends in decreasing the H band. After that, only the increasing trend of the O_3_ treatment for 6 min was different from the other treatments. It can be seen in Figure 7(b) that washing with distilled water for 4 min had the same trend with the control from day 4 to 12. Table 2 shows that for the HSV band, the ultrasonic treatment in distilled water for 6 min had a significant difference with the control in the S and V bands.

**FIGURE 8:**
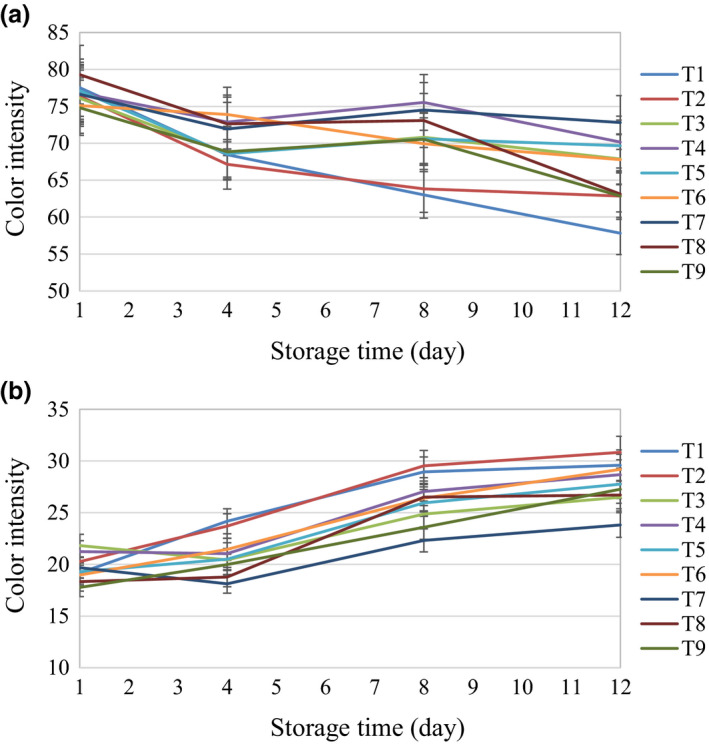
The changes in L*a*b* color space properties during the storage time (a) L* band, (b) b* band

Figure 8 and Table 2 reveal that based on the values of color bands in the L*a*b* space, the H_2_O_2_ treatment with distilled water for 6 min and ultrasonics with distilled water for 6 min had different trends with control, while other treatments were similar to the control. The first day mushrooms had a lighter color and higher brightness than other days. It can be said that the more a mushroom preserves its color brightness, the more suitable the treatment for use in button mushroom storage.

**FIGURE 9:**
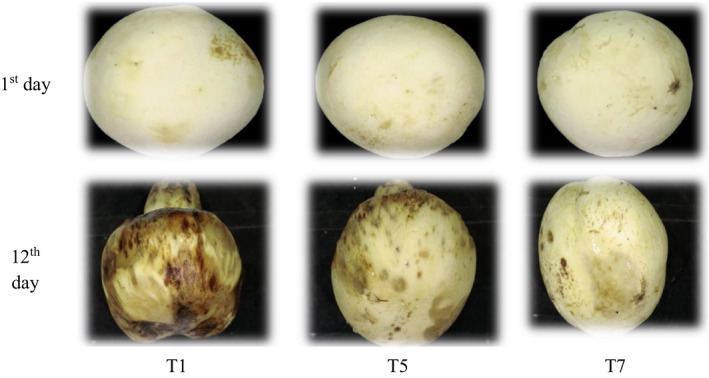
The visual appearance of the mushrooms under several treatments in the first and twelfth day of storage

According to the results obtained from RGB, HSV, and L*a*b* spaces, it can be concluded that the ultrasonic treatment in distilled water for 6 min can favorably affect the storage quality of the samples. Figure 9 shows several arbitrary mushroom samples studied under different treatments. This figure shows the appearance of the samples on the first day and the visual changes in their appearance after 12 days of storage. According to the figure, the positive effects of ultrasound for 6 min on the preservation of the color of the mushroom can be observed. After sonication, O_3_ treatment with distilled water for 6 min also had significant effects on the color preservation of the mushrooms.

### 3.2 Results of the total microbial counting

**FIGURE 10:**
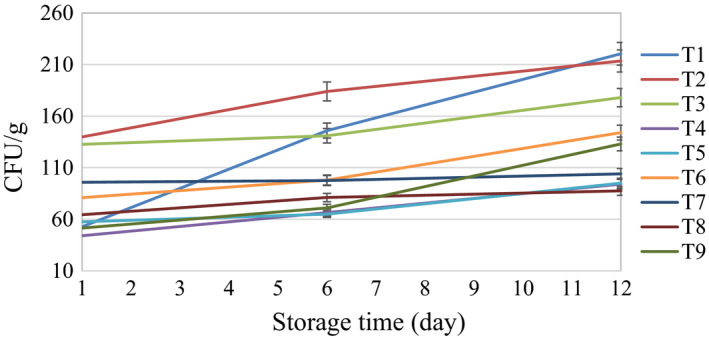
Total microbial counting of the samples during the storage time

Figure 10 shows the total microbial content of button mushroom samples during the 12 day storage at 4°C. According to the figure, the microbial contents of all samples under the treatments had an upward trend. At the end of the 12 day storage period, the lowest microbial content belonged to the ultrasonic treatment in distilled water for 6 min, which indicates that this treatment can significantly control the microbial load on the mushroom samples during the storage. Ultrasound reduces the microbial content on fruits and vegetables by destroying microorganisms and inactivating some enzymes without changing the quality properties of the products (de São José et al., 2014). Inactivation of microorganisms is the result of the formation, growth, and collapse of bubbles that are produced by the chemical and mechanical energy of the ultrasonic waves inside the cells of microorganisms. According to the figure, the highest amount of microbial content at the end of the storage period belonged to the control.

Several studies have reported the effect of H_2_O_2_ on reducing microbial contamination of cut mushrooms (Nerya et al., 2006). Jiang (2013) used alginate and nano‐silver particles to prevent the growth of bacteria. In the control samples, the number of mesophilic bacteria during 16 days of storage increased from 3.3 to 6.0 log CFU/g. Dalié et al. (2010) investigated the effects of essential oil on preventing browning and reducing the quality of button mushrooms. The results of this study on the number of total bacteria showed that the highest number of bacteria was observed in the control samples, and the essential oils of clove, cinnamon, and thyme had a great effect in preventing the growth of bacteria. The total number of bacteria during the period for the control samples increased from 4.3 to 7.0 log CFU/g. Jang and Moon (2011) reported that ultrasonic treatment combined with ascorbic acid inactivated many enzymes and reduced the microbial content, and therefore, increased the shelf life of apple cut pieces.

### 3.3 Results of the artificial neural networks

Having nine image and microbial properties as the input features of the model and storage time as the output, the performance of the ANN was investigated. To find the optimum network architecture for the prediction of the output, the network was trained by training set selected randomly from the dataset. Validation data were used to validate the quality of the proposed ANN model, where the stop criteria and weight reset were used to cope with underfitting/overfitting problems.

**FIGURE 11:**
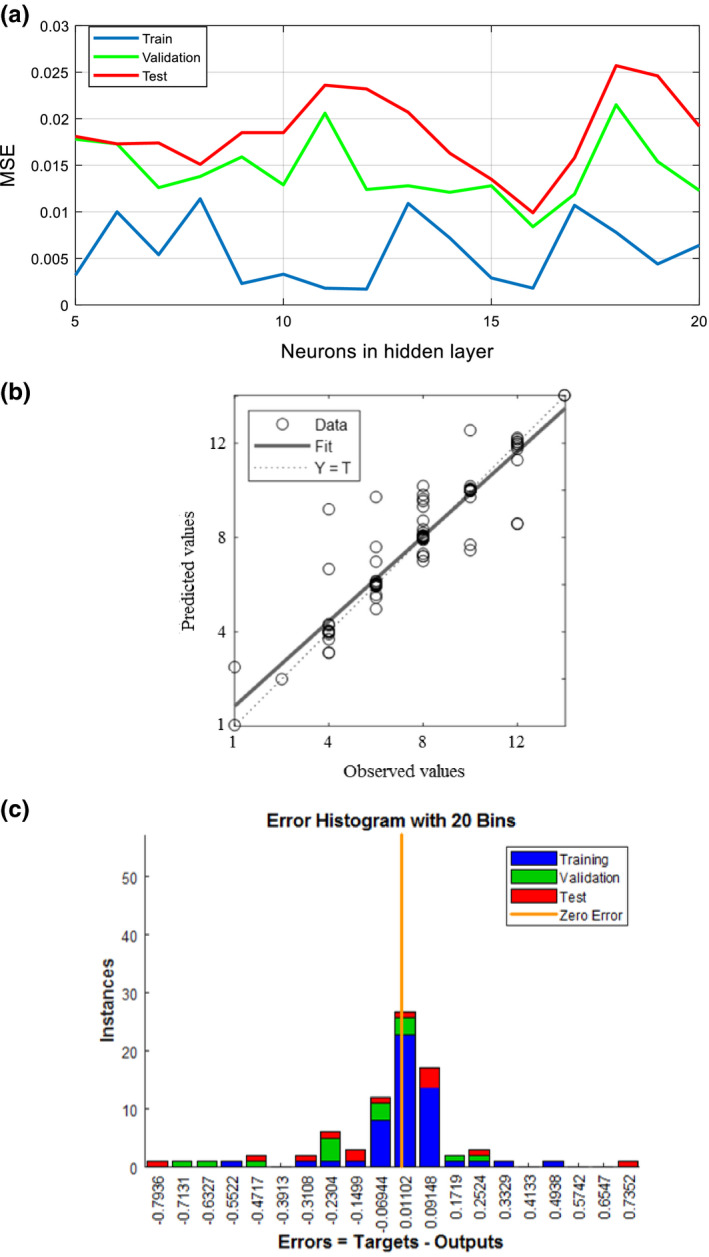
Performance of the ANN in the prediction of storage time, (a) *MSE* of the model as a function of the neurons in the hidden layer, (b) observed and predicted values of the storage time, (c) error of prediction

Figure 11(a) depicts the *MSE* values of the ANN in the prediction of the storage time. According to the figure, all the *MSE* values for train, validation, and test stages are obtained in the range of 0–0.03, which indicates the capability of the proposed ANN architecture for the prediction of network outputs.

One of the important parameters of the artificial neural network, which significantly affects the performance of this model, is the network architecture, that is, the size of the network hidden layer. The size of the hidden layer varied from 5 to 20 to obtain the optimum network. Figure 11(a) indicates that using 17 neurons in the hidden layer resulted in the minimum error (*MSE* = 0.011). However, there is no certain relationship between the accuracy and the number of nodes in the hidden layer of the ANN method. It was predictable (Asefpour Vakilian & Massah, 2018; Massah et al., 2019): by reducing the number of nodes in the hidden layer, weight, and bias of nodes’ sigmoid functions so vary to reduce the training error. Therefore, the performance of the network reduces significantly in predicting test data. On the other hand, an increase in the number of neurons in the hidden layer results in training sigmoid functions of the learner nodes with a few samples. In these conditions, the accurate prediction of test targets, particularly for out‐of‐range samples, will be accompanied by a significant error.

A comparison of observed and predicted data and the error values are shown in Figure 11(b),11(c), respectively, to indicate the closeness of the predicted and the measured value. The dashed line in each axis of Figure 11(b) represents the perfect result—predicted values—observed values. The solid line represents the best fit linear regression line between outputs and inputs. In general, the figure indicates a good fit between the outputs and the inputs.

## 4 DISCUSSION

Mushroom appearance characteristics are the most important parameter for consumer acceptance. After harvesting, the color of the button mushroom gradually turns from white to brown. The white matter of mushrooms decreases over time due to the effect of phenolic enzymes and the enzymatic formation of brown pigments by melanin. Research has shown that at temperatures below 14^°^C, antimicrobial solutions, as well as edible coatings, can reduce and control the process of color changes in the mushrooms. This undesirable browning is mainly due to enzyme oxidation, aging, and germ growth, resulting in loss of nutritional quality and lack of shelf life in fresh mushrooms (Oz et al., 2015). Although several types of enzymes are involved in the enzymatic color change of mushrooms, browning is mainly carried out by polyphenol oxidases, especially tyrosinase, and peroxidases (Kurtzman, 1997). Polyphenol oxidase can oxidize phenolic compounds to orthoquinones and cause browning of plant products, resulting in the inappropriate appearance and decrease of product quality. Therefore, to prevent enzymatic browning, polyphenol oxidase should be inactivated (Negishi and Ozawa., 2000).

Ultrasonic washing of agricultural products is a method to extend storage life. In recent years, there have been many advances in the use of ultrasound in food and agricultural processing, indicating its potential in the agricultural industry. Based on the results obtained from the RGB, HSV, and L*a*b* color spaces, it can be concluded that a 6 min treatment of ultrasonics is a reliable control method for the preservation of color value. This treatment was capable of maintaining the color of mushrooms during a 12 day storage period. It can also be concluded that O_3_ treatment with distilled water for 6 min after sonication had a significant effect on color preservation. Selecting the right ultrasonic treatment and the right treatment time causes the cavitation phenomenon in the product to occur correctly and to improve the postharvest characteristics of button mushrooms. Meanwhile, high ultrasound frequencies intensify the cavitation phenomenon and may release polyphenol oxidases and substrate exposure, leading to enzymatic browning reactions and increase browning (Shamaei et al., 2012). The findings of this study can be an appropriate guide in the industry for improving the storage quality of button mushrooms.

## AUTHOR CONTRIBUTION

**Maryam Ganjdoost:** Data curation (equal); Formal analysis (equal); Methodology (equal). **Mohammad Aboonajmi:** Investigation (equal); Project administration (equal); Supervision (equal); Writing‐original draft (equal). **Hossein Mirsaeedghazi:** Formal analysis (equal); Methodology (equal); Validation (equal). **Keyvan Asefpour Vakilian:** Methodology (equal); Software (equal); Writing‐review & editing (equal).
